# Hamilton County Medical Association

**Published:** 1837

**Authors:** 


### Art. VII.—Hamilton County Medical Association.

Pursuant of public notice, for a meeting of the physicians of Hamilton county, Ohio, at Carthage, on the 20th of May, 1837, the following gentlemen attended, viz :—A. Smith, J. W. Dunham, Wm. Goshorn, J. B. Rogers, J. P. Harrison, S. D. Gross, D. Drake, Wm. Wood, C. Dayton, W. V. Cowan, H. C. Mann, W. Mount, L. C. Rives, L. S. Pinkerton, T. Wright, J. B. Jewett, G. S. Close, H. Cox, J. G. Eson.

On motion, Dr. Asahel Smith, of Reading, was called to the Chair, and Dr. Lewis L. Pinkerton, of Carthage, appointed Secretary.

On motion of Dr. Pinkerton, it was

***Resolved***, That the Association shall consist of physicians, both of town and country, and that all who are in good standing may become members by subscribing the Constitution.

On motion, Drs. Drake, Harrison and Mount, were appointed a Committee to report on the organization of the Association, who, after retiring, reported a Constitution and system of By-Laws, which, after being amended, were adopted.

On motion of Dr. Harrison, it was

***Resolved***, That a Committee of three be appointed, to report, at the next meeting, a code of ethical rules, by which the physicians of this Association shall be regulated in their intercourse with each other, and with their patients. Whereupon, Drs. Harrison, Wright and Jewett, were appointed said Committee.

On motion of Dr. Drake, it was

*Resolved*, That the members of this Association, as far as may be consistent with their professional duties, will attend the second triennial meeting of the Medical Convention of Ohio, to be holden at Columbus, on the first Monday of January next.

On motion of Dr. Jewett, it was

*Resolved*, That it be the duty of each member of this Association to inform any physician or physicians residing near him of the formation of the Association, and invite him or them to attend its next meeting.

*Resolved*, That the President to be elected for the coming year, be requested to deliver an Inaugural Address at the next meeting.

Drs. Wright, Jewett and Mount, were appointed to read papers before the Association in July, and Drs. Cox, Wood, and Pinkerton, in October.

The meeting then proceeded to the election of officers, and the following was the result of the ballot :—

Dr. Smith, President.
Drs. Wright & Cox, V. Pr's, L. L. Pinkerton, Secretary.
Dr. Mount, Treasurer, Dr. Harrison, Orator.

By special invitation, Dr. Drake then addressed the meeting on the difficulties and discouragements of country physicians ; after which, the Association adjourned to meet in Carthage, on the third Saturday of July next, at 11 o'clock, A. M.

ASAHEL SMITH, President.

Lewis L. Pinkerton, Secretary.

